# Hospital-acquired sinusitis is a common cause of fever of unknown origin in orotracheally intubated critically ill patients

**DOI:** 10.1186/cc3805

**Published:** 2005-09-13

**Authors:** Arthur RH van Zanten, J Mark Dixon, Martine D Nipshagen, Remco de Bree, Armand RJ Girbes, Kees H Polderman

**Affiliations:** 1 Senior Consultant in Internal Medicine and Intensive Care, Department of Intensive Care, Gelderse Vallei Hospital, Ede, The Netherlands; 2 Senior Consultant in Anaesthesiology and Intensive Care, Department of Anesthesiology and Intensive Care, Norfolk and Norwich University Hospital, Norwich, UK; 3 Resident in Plastic Surgery, Hospital Hilversum, Hilversum, The Netherlands; 4 Professor of Intensive Care Medicine, Department of Intensive Care, VU University Medical Center, Amsterdam, The Netherlands; 5 Senior Consultant in Otolaryngology, Department of Otolaryngology/Head and Neck Surgery, VU University Medical Center, Amsterdam, The Netherlands; 6 Senior Consultant in Intensive Care, Department of Intensive Care, VU University Medical Center, Amsterdam, The Netherlands

## Abstract

**Introduction:**

Sinusitis is a well recognised but insufficiently understood complication of critical illness. It has been linked to nasotracheal intubation, but its occurrence after orotracheal intubation is less clear. We studied the incidence of sinusitis in patients with fever of unknown origin (FUO) in our intensive care unit with the aim of establishing a protocol that would be applicable in everyday clinical practice.

**Methods:**

Sinus X-rays (SXRs) were performed in all patients with fever for which an initial screening (physical examination, microbiological cultures and chest X-ray) revealed no obvious cause. All patients were followed with a predefined protocol, including antral drainage in all patients with abnormal or equivocal results on their SXR.

**Results:**

Initial screening revealed probable causes of fever in 153 of 351 patients (43.6%). SXRs were taken in the other 198 patients (56.4%); 129 had obvious or equivocal abnormalities. Sinus drainage revealed purulent material and positive cultures (predominantly *Pseudomonas *and *Klebsiella *species) in 84 patients. Final diagnosis for the cause of fever in all 351 patients based on X-ray results, microbiological cultures, and clinical response to sinus drainage indicated sinusitis as the sole cause of fever in 57 (16.2%) and as contributing factor in 48 (13.8%) patients with FUO. This will underestimate the actual incidence because SXR and drainage were not performed in all patients.

**Conclusion:**

Physicians treating critically ill patients should be aware of the high risk of sinusitis and take appropriate preventive measures, including the removal of nasogastric tubes in patients requiring long-term mechanical ventilation. Routine investigation of FUO should include computed tomography scan, SXR or sinus ultrasonography, and drainage should be performed if any abnormalities are found.

## Introduction

A large proportion of patients admitted to the intensive care unit (ICU) are likely to develop fever of unknown origin (FUO) at some point of their stay there. Many of these episodes are due to well-recognised hospital-acquired infections such as ventilator-associated pneumonia (VAP) and central venous catheter infections [1,2]. Various diagnostic strategies have been developed to handle such infectious complications in the ICU, many of which have been laid down in hospital or national guidelines [3,4]. However, the potential role of sinusitis as a source of hospital-acquired infections has been much less well studied. It is well recognised that sinusitis can occur as a complication of nasotracheal intubation; however, the incidence of sinusitis in patients after orotracheal intubation is unclear, and the data from the literature have been conflicting [5-8]. We therefore decided to assess the role of sinusitis as a hospital-acquired infection in mechanically ventilated and orotracheally intubated patients admitted to our ICU, in a prospective study using a rigorous protocol with predefined criteria for suspecting sinusitis.

Our aim was not only to assess the incidence of hospital-acquired sinusitis in patients with FUO but also to provide a practical protocol for diagnostic work-up and treatment that could be quickly implemented and easily applied in everyday clinical practice. Diagnostic and therapeutic procedures were therefore chosen in part on the basis of feasibility in daily clinical practice in the care of critically ill patients.

The three main imaging techniques available to establish a diagnosis of sinusitis are a standard sinus X-ray (SXR), ultrasound investigation, and computed tomography (CT) of the sinuses. Of these, a CT scan of the sinus cavities is unquestionably the most accurate and reliable procedure to establish the diagnosis of sinusitis. However, it would be highly impractical and costly to perform repeated CT scans on large numbers of ICU patients on a routine basis. In addition, transporting critically ill patients from the ICU to the radiology unit to perform a CT scan involves some risks [9-11]. A relatively new and promising development is the use of ultrasound as a diagnostic tool for sinusitis in the ICU setting, especially for the detection of maxillary sinusitis [12-15]; however, the reliability of this technique is strongly operator-dependent, and its sensitivity, especially in detecting frontal sinusitis, and overall specificity are relatively low [15-20]. Varonen and associates performed a meta-analysis of studies comparing SXR and ultrasound and reported that ultrasound was slightly less accurate than radiography when compared with the gold standard of sinus puncture [21]. Engels and associates [22] also concluded that, in spite of some limitations, sinus radiography rather than ultrasonography should still be viewed as the most reliable initial screening procedure for sinusitis. The most recent European Position Paper on Rhinosinusitis and Nasal Polyps recommends a combination of SXR followed by sinus puncture and aspiration as the diagnostically most accurate procedure [23].

It should be pointed out that most of these studies were not performed in mechanically ventilated ICU patients, and some studies have suggested that ultrasound has a higher sensitivity and specificity in the ICU setting. However, ultrasound has not so far been widely adopted as a first-line diagnostic tool for sinusitis, and most ICUs use plain SXRs as a first-line screening tool. We therefore chose SXR as our initial screening technique.

## Methods

### Patients

The study was performed in accordance with guidelines laid down by the hospital ethics committee. All mechanically ventilated adult patients admitted to the surgical wing of our intensive care department during the 18-month study period who spent more than 48 hours in the ICU and who developed fever during their ICU stay were included in the study. Inclusion criteria were as follows: age 18 to 80 years; core temperature 38.5°C (measured in oesophagus, bladder or rectum); not admitted for infections or, if infection was the primary reason for admission, infection treated and temperature normalised for at least 72 hours before recurrence of FUO. At the time of our study, gastric tubes were inserted nasally in most patients. Sedation and analgesia were given in the context of a nurse-driven sedation protocol using the Ramsey score to guide levels of sedation. Exclusion criteria included severe head and facial injuries, skull fractures and immunocompromised patients.

FUO was defined as follows: the cause of fever not immediately clear; the patient was not admitted because of fever or sepsis, or the patient had recovered from one or more previous septic episodes or infections. This means that some patients were admitted with, for example, abdominal sepsis, and developed sinusitis in the course of their admission. Such patients were eligible for inclusion in our study.

### Protocol

According to our protocol all patients who developed fever first underwent routine analysis, which included physical examination, drawing of blood cultures and analysis for white blood cell count, and a chest X-ray. Central lines were changed if they had been in place for 1 week or more, or if there were any signs of local infection [2].

An SXR was taken if a cause of fever did not become clear from the above mentioned analysis. An SXR was also taken if a cause of fever was found on routine analysis but when fever persisted for more than 48 hours in spite of antibiotic therapy to exclude sinusitis as the primary cause of fever and/or a contributing factor.

SXRs were taken in two directions, the straight anterior–posterior view (Caldwell view) and the lateral view, using portable devices in the ICU. Additional X-rays were taken if the first X-rays were difficult to interpret, in accordance with our routine for radiodiagnostic procedures [24]. Interpretations were made by the attending physician and confirmed by an independent radiologist. Three categories were used: abnormal (clouded sinuses with fluid), equivocal and normal.

Patients with an abnormal SXR were treated by an ear, nose and throat (ENT) surgeon with diagnostic and therapeutic antral sinus tap and drainage [22,23]. The procedure had to be performed as soon as possible, but a maximum interval of 12 hours was allowed if there was a need for correction of coagulopathy. To prevent accidental contamination the nares were swabbed with chlorhexidine before puncture. Macroscopic inspection of the aspirate was performed by the ENT surgeon using four categories: pus, purulent, bloody and clear. In all cases samples were taken for both aerobic and anaerobic cultures. Cultures were performed using semi-quantitative methods (no growth, 0 colonies; +, 1 to 10 colonies; ++, 10 to 100 colonies; +++, more than 100 colonies), with ++ or +++ being regarded as positive and 0 or + as negative. Repeated drainage could be scheduled at the discretion of the attending ENT surgeon on clinical grounds. Patients with equivocal and normal results on SXR were followed up. In patients with equivocal results a repeat SXR was made 48 hours later unless the fever had resolved or another cause of fever had been found. In patients with normal SXRs no repeat was indicated except at the discretion of the attending physician.

Final diagnosis for cause of fever in all 351 patients was based on blood, sputum and sinus cultures as applicable, chest X-rays and on clinical criteria (normalisation of temperature after removal of the central line, or after sinus drainage, response to antibiotic treatment, and so on).

Statistical analyses were performed with Student's *t*-test for unpaired groups. Results are expressed as means ± SD. Statistical significance was accepted at *P *< 0.05.

## Results

**Figure 1:**
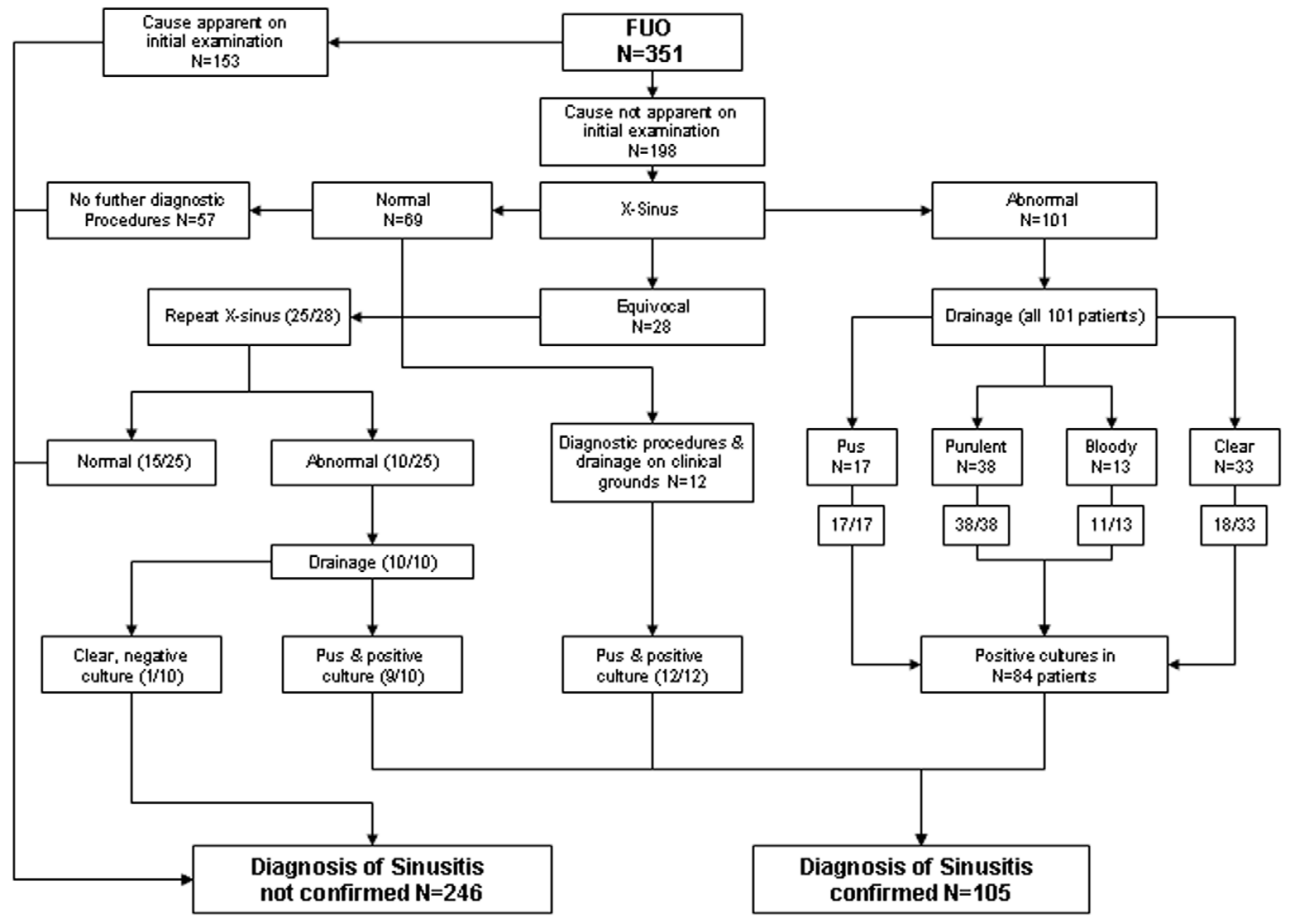
Flowchart depicting the organisation of our study in patients with fever of unknown origin (FUO), as well as the diagnostic work-up and culture results.

The results are summarised in Fig. 1.

During a period of 15 months a total of 351 patients met the initial inclusion criteria. In 153 patients a probable cause of fever was found on routine analysis. Therefore 198 patients met the criteria for SXR. Patient data and the results of these X-rays are shown in Table 1.

On the basis of the results of the SXR, sinus drainage was first performed in those patients with evident abnormalities (*n *= 101). Drainage was performed in 98 of these 101 patients within 12 hours (mean 2.05 ± 5.7 hours). Twenty-four patients had been given platelets or fresh frozen plasma before the procedure. In three patients the procedure was delayed for a longer period because of the use of anticoagulants and/or platelet aggregation inhibitors. In these patients the procedure was performed within 48 hours.

Repeat drainage was performed in 41 patients after an average period of 52 ± 38 hours.

The initial (macroscopic) interpretation of the material obtained during the draining procedure by the ENT surgeon was pus in fluid from 17 of 101 patients (17%), purulent in 38 (38%), bloody in 13 (13%) and clear in 33 (33%). Culture results of initial sinus drainage are shown in Table 2. Many patients had more than one microorganism cultured from the sinus fluid. A total of 140 microorganisms were cultured from 84 of these 101 sinus drainage fluids (84%). All cultures that had been deemed as pus or purulent on macroscopic evaluation turned out positive for pathogenic microorganisms. However, bacteria were also cultured from a substantial proportion (18 of 33 (55%)) of the fluids that had been deemed clear on microscopic inspection. The cultured pathogens are listed in Table 2. The most predominant microorganisms in the sinus fluids were Gram-negative pathogens such as *Pseudomonas *and *Klebsiella *species.

Of the 28 patients with indeterminate or equivocal results on the initial SXR, a repeat SXR was performed in 25 patients. Ten (40%) now had obvious abnormalities, and drainage was performed. Of these the diagnosis of sinusitis was confirmed in 9 patients. Of the 69 patients with an initially normal SXR, sinus drainage was nevertheless performed in the subsequent 72-hour period in 12 patients on clinical grounds (*n *= 2), repeat SXR (*n *= 2) and following CT scan (*n *= 8). The diagnosis was confirmed by drainage and cultures in all 12 of these patients. Thus, a total of 21 cases (22%) of microbiological sinusitis were subsequently found in the group of 97 patients who initially had equivocal or normal findings on SXR.

On the basis of these clinical, radiological and microbiological criteria we evaluated the final diagnoses in all 351 patients with FUO initially included in our study. The results are shown in Tables 3 and 4.

## Discussion

The results of our study demonstrate that sinusitis is a frequently occurring hospital-acquired infection in the ICU. Sinusitis was initially diagnosed in 84 of 351 (24%) patients with FUO, and in an additional 21 patients who had equivocal or normal findings on initial SXR, giving a total of 105 of 351 patients (30%). This underestimates the true incidence because SXRs were not taken in 153 patients who had obvious other causes of fever on initial screening, some of whom might also have had sinusitis.

Sinusitis was the sole cause of fever in 57 patients (16%) and one of several causes (for example sinusitis and purulent bronchitis) in 48 patients (14%). Pathogenic microorganisms were cultured not only when material obtained by antral sinus puncture was classified as 'purulent' but also in more than half of the patients whose puncture material was less suspect on macroscopic examination.

Previous studies on sinusitis in orotracheally intubated patients have reported a lower incidence of sinusitis than was observed in our study, ranging from 2% to 7.7% [5,7,25,26]. There are several possible reasons for this. First, the rate of antibiotic resistance in the Netherlands is low, and antibiotics are used relatively sparingly. This might have reduced the likelihood of undetected sinusitis being concomitantly treated because patients were receiving antibiotics for other infections [25,27]. Second, our patients were more severely ill than patients included in the previous studies, as demonstrated by a high average severity of disease score (Acute Physiology and Chronic Health Evaluation (APACHE)-II score of 21 ± 6.8 in our study, compared with Simplified Acute Physiology Score (SAPS) II scores of 12 ± 4.5 [5] and 11.0 ± 3.5 [7]; other studies reported no severity scores). Third, risk factors for sinusitis such as sedation and nasogastric tube feeding were present more frequently in our patients, perhaps because of the greater severity of disease.

Of the positive cultures in our patients, 77% contained Gram-negative pathogens. This rate is higher than reported in previous studies, in which about 50% of cultured pathogens were Gram-positive [8,25,28,29]. This might be explained by differences in case mix, severity of illness and length of ICU stay, as well as effects of previous antibiotic treatment on the patients' microflora [25,28,29].

Our study has some limitations. The diagnosis was based on abnormal findings on SXR and positive microbiological cultures obtained after antral drainage. However, SXRs cannot accurately distinguish purulent sinusitis from sterile fluids, so abnormal SXRs may overestimate the incidence of sinusitis [8,25]. Moreover, even positive microbiological cultures may not prove clinically relevant sinusitis, because they may indicate colonisation rather than actual infection. We tried to circumvent these problems by classifying only cultures with more than 10 colonies of bacteria as positive and by basing our diagnosis on a combination of radiological abnormalities, positive cultures, and clinical response to therapeutic measures such as drainage and targeted antibiotic treatment. We are therefore confident that our results accurately reflect the true incidence of sinusitis.

Early detection and treatment is important because delays can lead to the development of VAP, sepsis, and life-threatening complications such meningitis, mastoiditis, intra-cranial abscesses and venous thrombosis of the sinus cavernosus [24,30,31]. Early treatment of sinusitis may significantly reduce the risk of VAP and perhaps also ICU mortality [8,32].

The results of our microbiological cultures underline the close link between sinusitis and the development of VAP. Of 105 patients in whom positive sinus cultures were obtained, the same microorganisms were cultured from bronchotracheal aspirates in 40% of cases (*n *= 42). In some patients we were able to demonstrate that positive sinus cultures preceded positive cultures from the lungs, strongly suggesting that sinusitis can lead to infections of the lower airway. Others reported similar observations; for example, Holzapfel and co-workers found that the early detection and treatment of hospital-acquired sinusitis could prevent the occurrence of VAP and reduce mortality in nasotracheally intubated ICU patients [33].

Bacteraemia with the same microorganism as that cultured from the sinus occurred in 12 patients; in five patients the microorganism causing bacteraemia was cultured only from the blood and the sinus, making sinusitis the most likely cause of bacteraemia. However, no definite conclusions about cause and effect can be drawn because bacteraemia can also lead to sinusitis, with bacterial colonisation of sinus fluids following bacteraemia [30,34].

Various mechanisms might explain the high incidence of sinusitis in ICU patients. The first is anatomical. The paranasal sinuses secrete mucus that flows to the natural ostia located posteriorly towards the nasopharynx; this flow can be blocked by infection, inflammation, anatomic abnormalities or the presence of foreign material such as nasotracheal intubation tubes. Even tubes with smaller diameters (such as nasogastric feeding and suction tubes) can cause significant obstruction in the normal flow of sinus fluids, leading to an increased risk of bacterial colonisation and development of hospital-acquired sinusitis [25,28]. The presence of nasogastric tubes has been linked to a significant increase in the risk for sinusitis in mechanically ventilated patients (odds ratio 14.1, 95% confidence interval 1.7 to 117) [25]. Another important risk factor is the use of sedatives (odds ratio 15.9, 95% confidence interval 1.9 to 133.5) [25]. Underlying mechanisms may include the suppression of normal cleansing mechanisms such as coughing, sneezing and nose-blowing, because of sedation and analgesia; in addition, immobility precludes positional changes that improve mucous drainage under normal circumstances [24]. Remaining in a recumbent position can increase nasal congestion and obstruction of the ostia of the maxillary sinuses. This problem may be compounded by the positive inspiratory and end-expiratory pressure in ventilated patients, which also induces an increase in central venous pressure [6,35]. In addition, critically ill patients recovering from earlier episodes of sepsis may develop relative immune suppression, so-called immunoparalysis [36].

ICU patients are often unable to communicate, and complaints related to sinusitis may go unnoticed by the medical and nursing staff. Patients may have a 'runny nose', or discharge of purulent material from the nasal cavity. However, this is seen in only 27% of cases [37]. Thus elevations in white blood cell count and/or FUO may be the first presenting symptoms [24].

In theory, the use of imaging modalities such as CT scans [24,38,39] and B-mode ultrasound [12-14] could improve the diagnostic yield. As discussed above, the CT scan should be regarded as the gold standard for the diagnosis of sinusitis. Unfortunately, CT scans are not easily performed in the ICU setting, meaning that the patient has to be transported to the department of radiology for this procedure. These in-hospital transports can be risky [9,10,40,41]. The potential benefits in establishing or confirming the diagnosis should therefore be weighed against the risks of transport. The development of mobile CT scans for use at bedside would significantly reduce these problems; however, such devices are not yet available in most hospitals.

Some authors have suggested that ultrasound may provide a good or even better alternative to SXR for detecting sinusitis at bedside in critically ill patients [12,13]. However, ultrasound is not yet widely used for this purpose in routine clinical practice. Moreover, its diagnostic accuracy depends on the experience of the operator, and the costs are higher than for SXR. In addition, the literature comparing diagnostic yields of ultrasound and SXR provides conflicting results [12,13,21,22,42]. Our study was not designed to compare the two techniques; we based our choice mainly on the fact that ultrasound is not yet widely used to detect sinusitis in the ICU setting, and on our pre-existing clinical protocols. It seems unlikely that use of ultrasound for initial screening would have significantly affected our results; at best it could have increased our diagnostic yield, further strengthening our observation that sinusitis is a frequent cause of FUO in ICU patients. In addition, about 85% of the cases of hospital-acquired sinusitis associated with mechanical ventilation involve the maxillary sinuses [6]. As conventional SXRs are most reliable in detecting maxillary sinusitis (in comparison with frontal and ethmoidal sinusitis) we feel that SXRs remain the most practical diagnostic tool, with an acceptable sensitivity for detecting sinusitis in the ICU setting. Hospitals favouring ultrasonography as initial screening method could easily adapt our protocol, replacing SXR by ultrasound. The CT scan remains the radiological gold standard in the diagnosis of sinusitis.

On the basis of the results of our study we recommend that hospital-acquired sinusitis be considered in all patients with FUO in the ICU in whom a cause of fever is not immediately apparent from initial examination and chest X-ray. SXR or ultrasound or (if possible) a CT scan should be included in the diagnostic work-up, and sinus puncture with drainage should be performed in case of abnormal or equivocal findings. In our study all procedures were performed at thr bedside; 40% of patients with confirmed sinusitis required repeat drainage, but no patients required more than two procedures.

All nasal tubes should be removed if sinusitis is suspected; antibiotics should be started empirically or based on Gram staining, and adjusted for final culture results. In most patients temperature normalises within 48 hours [37]; this was also observed in our study. Radiological signs of sinusitis clear more slowly but should disappear within ±1 week [43].

The results of our study have led to the implementation of several measures to reduce the incidence of sinusitis. First, nasogastric tubes are no longer used in intubated patients unless it is expected that the endotracheal tube can be removed within 24 hours. Gastric tubes in all other patients are now inserted through the mouth. Second, patients intubated for ≥24 hours now routinely receive topical administration of saline 0.9% and/or decongestants such as xylometazoline drops in the nasal cavities. Thirdly, the nursing staff keeps a far more rigorous watch for signs of purulent nasal discharge in all patients, and diagnostic procedures such as X-sinus are performed if such discharge is observed. Finally, the routine diagnostic work-up in patients who develop fever in the ICU now includes an SXR. Drainage (both as a diagnostic and therapeutic tool) takes place in all patients with clear or equivocal signs of sinusitis. Topical decongestants are used to reduce oedema and facilitate drainage. In patients with clear SXR in whom no other diagnosis is established, SXR is repeated after 48 hours. These measures have led to a marked reduction in the incidence of sinusitis in our ICU.

## Conclusion

Hospital-acquired sinusitis is a frequent cause of FUO in orotracheally intubated and mechanically ventilated critically ill patients. ICU physicians should be aware of the numerous risk factors for sinusitis simultaneously present in ICU patients and take appropriate preventive measures. We recommend including an SXR in the routine work-up for FUO in all ICU patients; drainage should take place if SXR reveals clouding, and should also be considered if the SXR is equivocal or difficult to interpret. A normal SXR does not rule out sinusitis, and when in doubt drainage or additional diagnostic procedures such as CT scan should be performed.

## Key messages

• 	Sinusitis is a frequent cause of FUO in the ICU (in this study it was the sole cause in 16% and a contributing factor in 13% of patients with FUO).

• 	Bacterial colonisation of the sinuses often precedes the development of bronchitis and VAP; sinusitis may be a frequent cause of hospital-acquired bronchitis and VAP.

• 	Diagnostic work-up of FUO should include an SXR, ultrasound or CT scan; drainage should be performed if any abnormalities are found.

• 	Physicians treating critically ill patients should be aware of the high risk of sinusitis and take appropriate preventive measures, including the removal of nasogastric tubes in patients requiring long-term mechanical ventilation.

## Abbreviations

CT = computed tomography; ENT = ear, nose and throat; FUO = fever of unknown origin; ICU = intensive care unit; SXR = sinus X-ray; VAP = ventilator-associated pneumonia.

## Competing interests

The author(s) declare that they have no competing interests.

## Authors' contributions

KHP, JMD and ARG designed and coordinated the study. AvZ, RdB, JMD and KHP were involved in the collection, statistical analysis and interpretation of the data. MDN performed literature analysis and assisted in the data collection. AvZ and KHP drafted and revised the manuscript. All authors read and approved the final manuscript.

## Figures and Tables

**Table 1:** Epidemiological data, results of SXR and macroscopic evaluation of sinus fluids obtained by antral puncture

Parameter	Value
Patient data (*n *= 351)	
Sex (M:F)	193:158
Age (mean ± SD)	59 ± 21.2
APACHE II score (mean ± SD)	21 ± 6.8
ICU LOS (days) at diagnosis (mean ± SD)	5.9 ± 5.7
Results of sinus X-rays in patients with FUO (*n *= 198)	
Sinus X-ray abnormal (two directions)	101 (51%)
Sinus X-ray equivocal	28 (14%)
Sinus X-ray normal	69 (35%)
Macroscopic evaluation of sinus fluid (*n *= 101)	
Pus	17 (17%)
Purulent	38 (38%)
Bloody	13 (13%)
Clear	33 (33%)

APACHE, Acute Physiology and Chronic Health Evaluation; FUO, fever of unknown origin; UCI LOS, length of stay in the intensive care unit.

**Table 2:** Results of sinus fluid cultures of patients with gross abnormalities in their initial sinus X-ray

Bacterium	Sinus fluid	Same MO cultured from tracheal aspirate	Same MO cultured from blood
*Pseudomonas aeruginosa*	32	22	8
*Klebsiella oxytoca*	5	3	1
*Klebsiella pneumoniae*	17	10	2
*Enterococcus faecalis*	10	2	1
*Enterobacter cloacae*	12	4	0
*Escherichia coli*	16	8	1
*Staphylococcus aureus*	8	3	1
Gram-positive mixture	11	-	-
Gram-negative mixture	14	-	-
Other^a^	15	8	1
Total	140	60	15

There were 101 patients with gross abnormalities in their initial sinus X-ray. Positive cultures were obtained in 84 patients, with 140 different types of microorganisms (MOs). Columns 3 and 4 show positive results of the same microorganisms (MOs) cultured from tracheal aspirate and blood, cultured in the period between 24 hours before and 48 hours after sinus drainage.

^a^Other pathogens included anaerobic bacteria (such as *Bacteroides *sp.) and fungi (*Candida *sp.).

**Table 3:** Initial diagnosis for fever of unknown origin in mechanically ventilated patients in intensive care unit

Cause of fever	Sole cause	One of multiple causes
Central line infection	43	1
Upper respiratory tract infection/pneumonia^a^	93	42
Sinusitis	45	39
Abdominal focus	5	3
Other^b^	2	1
Unknown		121
Total	188	86

Initial diagnosis was performed after initial screening with physical examination and chest X-ray in all 351 patients, sinus X-ray in 198 patients and sinus drainage in 98 patients; cultures were not yet available. All patients had fever and leucocytosis. ^a^Purulent tracheobronchial aspirate with cultures positive for pathogenic microorganisms, combined with new or progressive pulmonary infiltrates on chest X-ray; ^b^other causes of fever included meningitis, phlebitis and deep venous thrombosis.

**Table 4:** Final diagnosis for FUO at ICU discharge, with final results of all cultures known

Cause of fever	Sole cause	One of multiple causes^a^
Central line infection	44	11
Upper respiratory tract infection/pneumonia	132	58
Sinusitis	57	48
Abdominal focus	8	16
Other^b^	12	28
Unknown		46
Total	253	161

See also Fig. 1. ^a^Most patients with more than one cause of fever had sinusitis and bronchitis/pneumonia; ^b^other causes of fever included meningitis (not related to sinusitis), phlebitis and deep venous thrombosis.
